# 2-*p*-Tolyl-4,5-dihydro-1*H*-imidazole

**DOI:** 10.1107/S1600536809008125

**Published:** 2009-03-11

**Authors:** Reza Kia, Hoong-Kun Fun, Hadi Kargar

**Affiliations:** a X-ray Crystallography Unit, School of Physics, Universiti Sains Malaysia, 11800 USM, Penang, Malaysia; b Department of Chemistry, School of Science, Payame Noor University (PNU), Ardakan, Yazd, Iran

## Abstract

In the mol­ecule of the title compound, C_10_H_12_N_2_, the six- and five-membered rings are almost co-planar, forming a dihedral angle of 3.56 (8)°. In the crystal structure, neighbouring mol­ecules are linked together by inter­molecular N—H⋯N hydrogen bonds into one-dimensional infinite chains along the *c* axis. The crystal structure, is further stabilized by weak inter­molecular C—H⋯π and π–π stacking [centroid–centroid distance = 3.8892 (9) Å] inter­actions.

## Related literature

For bond-length data, see: Allen *et al.* (1987 ▶). For hydrogen-bond motifs, see: Bernstein *et al.* (1995 ▶). For related structures and syntheses, see, Stibrany *et al.* (2004 ▶); Kia *et al.*, 2008 ▶, 2009 ▶). For applications of imidazoline derivatives, see, for example: Blancafort (1978 ▶); Chan (1993 ▶); Vizi (1986 ▶); Li *et al.* (1996 ▶); Ueno *et al.*, (1995 ▶); Corey & Grogan (1999 ▶). For the stability of the temperature controller used for the data collection, see: Cosier & Glazer (1986 ▶).
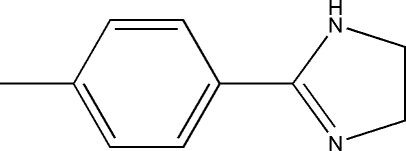


## Experimental

### 

#### Crystal data

C_10_H_12_N_2_
*M* *_r_* = 160.22
Monoclinic, 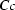

*a* = 5.1134 (1) Å
*b* = 16.4020 (4) Å
*c* = 10.1712 (2) Å
β = 94.293 (1)°
*V* = 850.66 (3) Å^3^
*Z* = 4
Mo *K*α radiation
μ = 0.08 mm^−1^
*T* = 100 K
0.47 × 0.12 × 0.09 mm

#### Data collection

Bruker SMART APEXII CCD area-detector diffractometer
Absorption correction: multi-scan (*SADABS*; Bruker, 2005 ▶) *T*_min_ = 0.883, *T*_max_ = 0.993
8503 measured reflections
1423 independent reflections
1338 reflections with *I* > 2˘*I*)
*R*_int_ = 0.031

#### Refinement

*R*[*F* ^2^ > 2σ(*F* ^2^)] = 0.037
*wR*(*F* ^2^) = 0.102
*S* = 1.08
1423 reflections
114 parameters
2 restraints
H atoms treated by a mixture of independent and constrained refinement
Δρ_max_ = 0.33 e Å^−3^
Δρ_min_ = −0.21 e Å^−3^

### 

Data collection: *APEX2* (Bruker, 2005 ▶); cell refinement: *SAINT* (Bruker, 2005 ▶); data reduction: *SAINT*; program(s) used to solve structure: *SHELXTL* (Sheldrick, 2008 ▶); program(s) used to refine structure: *SHELXTL*; molecular graphics: *SHELXTL*; software used to prepare material for publication: *SHELXTL* and *PLATON* (Spek, 2009 ▶).

## Supplementary Material

Crystal structure: contains datablocks global, I. DOI: 10.1107/S1600536809008125/at2738sup1.cif

Structure factors: contains datablocks I. DOI: 10.1107/S1600536809008125/at2738Isup2.hkl

Additional supplementary materials:  crystallographic information; 3D view; checkCIF report

## Figures and Tables

**Table 1:** Hydrogen-bond geometry (Å, °)

*D*—H⋯*A*	*D*—H	H⋯*A*	*D*⋯*A*	*D*—H⋯*A*
N1—H1*N*1⋯N2^i^	0.87 (3)	2.06 (3)	2.9224 (18)	170 (2)
C10—H10*B*⋯*Cg*1^ii^	0.96	2.88	3.8110 (16)	163

Symmetry codes: (i) 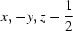; (ii) 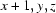. *Cg*1 is the cetroid of the N1/C2/C1/N2/C3 ring.

**Fig. 1.**
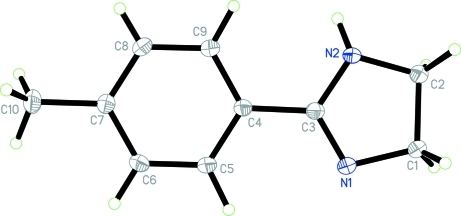
The molecular structure of (I) with atom labels and 50% probability ellipsoids for non-H atoms.

**Fig. 2.**
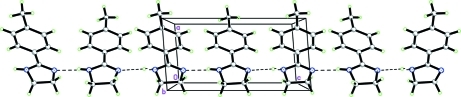
The crystal packing of (I), viewed down the b-axis showing a 1-D infinite chain along the c-axis by intermolecular N—H···N interactions. The intermolecular interactions are shown as dashed lines.

## supplementary crystallographic information

### Comment

Imidazoline derivatives are of great importance because they exhibit significant biological and pharmacological activities such as antihypertensive (Blancafort 1978), antihyperglycemic (Chan 1993), antidepressive (Vizi 1986), antihypercholesterolemic (Li *et al.*, 1996) and antiinflammatory (Ueno *et al.*, 1995). These compounds are also used as catalysts and synthetic intermediates in some organic reactions (Corey & Grogan 1999). With regards to the important applications of imidazolines, herein we report the crystal structure of the title compound, (I).

In the title compound (I, Fig. 1), bond lengths (Allen *et al.* 1987) and angles are within the normal ranges and are comparable with the related structures (Stibrany *et al.* 2004; Kia *et al.*, 2008, 2009). The molecule is almost planar with a maximum deviation from the mean plane of the molecule for C2 atom being -0.176 (19) Å. The six- and five-membered rings are twisted from each other, forming the dihedral angle of 3.56 (8)°. The interesting feature of the crystal structure is the short C2···C10^i^ contact [3.368 (2) Å; (i) 1 + *x*, *y*, *z*], which is shorter than the sum of the van der Waals radius of carbon atom. In the crystal structure, neighbouring molecules are linked together by intermolecular N—H···N hydrogen bonds into 1-D infinite chains along the *c* axis (Table 1, Fig. 2). The crystal structure is further stabilized by weak intermolecular π-π stacking [*Cg1*···*Cg2*^iii^ = 3.8892 Å; (iii) -1 + *x*, *y*, *z*] and C—H···π interactions (*Cg1* and *Cg*2 are the centroids of the N1/C2/C1/N2/C3-imidazoline and the benzene rings, respectiverly).

### Experimental

The synthetic method was based on the previous work (Stibrany *et al.* 2004), except that 10 mmol of 4-methyl cyanobenzene and 40 mmol of ethylenediamine was used. Single crystals suitable for *X*-ray diffraction were obtained by evaporation of an methanol solution at room temperature.

### Refinement

The N-bound hydrogen was located from the difference Fourier map are refined freely (see Table. 1). The rest of the hydrogen atoms were positioned geometrically with a riding approximation model with C—H = 0.93–0.97 Å and *U*_iso_(H) = 1.2 & 1.5 *U*_eq_(C). A rotating group model was applied for the methyl group. The 1120 Friedel pairs were merged before final refinement as there is not sufficient anomalous dispersion to determine the absolute structure.

### Crystal data

**Table:**

C_10_H_12_N_2_	*F*(000) = 344
*M**_r_* = 160.22	*D*_x_ = 1.251 Mg m^−^^3^
Monoclinic, *C**c*	Mo *K*α radiation, λ = 0.71073 Å
Hall symbol: C -2yc	Cell parameters from 3821 reflections
*a* = 5.1134 (1) Å	θ = 2.5–31.5°
*b* = 16.4020 (4) Å	µ = 0.08 mm^−^^1^
*c* = 10.1712 (2) Å	*T* = 100 K
β = 94.293 (1)°	Needle, colourless
*V* = 850.66 (3) Å^3^	0.47 × 0.12 × 0.09 mm
*Z* = 4	

### Data collection

**Table:**

Bruker SMART APEXII CCD area-detector diffractometer	1423 independent reflections
Radiation source: fine-focus sealed tube	1338 reflections with *I* > 2˘*I*)
graphite	*R*_int_ = 0.031
φ and ω scans	θ_max_ = 31.5°, θ_min_ = 2.5°
Absorption correction: multi-scan (*SADABS*; Bruker, 2005)	*h* = −7→7
*T*_min_ = 0.883, *T*_max_ = 0.993	*k* = −24→24
8503 measured reflections	*l* = −14→14

### Refinement

**Table:**

Refinement on *F*^2^	Primary atom site location: structure-invariant direct methods
Least-squares matrix: full	Secondary atom site location: difference Fourier map
*R*[*F*^2^ > 2σ(*F*^2^)] = 0.037	Hydrogen site location: inferred from neighbouring sites
*wR*(*F*^2^) = 0.102	H atoms treated by a mixture of independent and constrained refinement
*S* = 1.08	*w* = 1/[σ^2^(*F*_o_^2^) + (0.0699*P*)^2^ + 0.0868*P*] where *P* = (*F*_o_^2^ + 2*F*_c_^2^)/3
1423 reflections	(Δ/σ)_max_ < 0.001
114 parameters	Δρ_max_ = 0.33 e Å^−^^3^
2 restraints	Δρ_min_ = −0.21 e Å^−^^3^

### Special details

**Table:**

Experimental. The crystal was placed in the cold stream of an Oxford Cyrosystems Cobra open-flow nitrogen cryostat (Cosier & Glazer, 1986) operating at 100.0 (1) K.
Geometry. All e.s.d.'s (except the e.s.d. in the dihedral angle between two l.s. planes) are estimated using the full covariance matrix. The cell e.s.d.'s are taken into account individually in the estimation of e.s.d.'s in distances, angles and torsion angles; correlations between e.s.d.'s in cell parameters are only used when they are defined by crystal symmetry. An approximate (isotropic) treatment of cell e.s.d.'s is used for estimating e.s.d.'s involving l.s. planes.
Refinement. Refinement of *F*^2^ against ALL reflections. The weighted *R*-factor *wR* and goodness of fit *S* are based on *F*^2^, conventional *R*-factors *R* are based on *F*, with *F* set to zero for negative *F*^2^. The threshold expression of *F*^2^ > σ(*F*^2^) is used only for calculating *R*-factors(gt) *etc*. and is not relevant to the choice of reflections for refinement. *R*-factors based on *F*^2^ are statistically about twice as large as those based on *F*, and *R*- factors based on ALL data will be even larger.

### Fractional atomic coordinates and isotropic or equivalent isotropic displacement parameters (Å^2^)

**Table:**

	*x*	*y*	*z*	*U*_iso_*/*U*_eq_	
N2	−0.2547 (3)	−0.01211 (8)	1.08200 (13)	0.0175 (3)	
N1	−0.2444 (3)	−0.02899 (8)	0.86191 (12)	0.0189 (3)	
C1	−0.4382 (3)	−0.07930 (9)	1.04580 (15)	0.0190 (3)	
H1A	−0.6091	−0.0684	1.0774	0.023*	
H1B	−0.3728	−0.1303	1.0837	0.023*	
C2	−0.4570 (3)	−0.08371 (9)	0.89368 (15)	0.0181 (3)	
H2A	−0.4279	−0.1388	0.8631	0.022*	
H2B	−0.6256	−0.0644	0.8561	0.022*	
C3	−0.1632 (3)	0.01280 (8)	0.97337 (13)	0.0142 (3)	
C4	0.0262 (3)	0.08065 (8)	0.96924 (15)	0.0141 (2)	
C5	0.1072 (3)	0.12199 (9)	1.08498 (14)	0.0189 (3)	
H5A	0.0404	0.1069	1.1640	0.023*	
C6	0.2870 (3)	0.18556 (9)	1.08343 (15)	0.0201 (3)	
H6A	0.3393	0.2125	1.1615	0.024*	
C7	0.3900 (3)	0.20952 (8)	0.96598 (14)	0.0171 (3)	
C8	0.3049 (3)	0.16917 (9)	0.85050 (15)	0.0216 (3)	
H8A	0.3690	0.1851	0.7712	0.026*	
C9	0.1253 (3)	0.10529 (9)	0.85124 (15)	0.0202 (3)	
H9A	0.0712	0.0790	0.7729	0.024*	
C10	0.5915 (3)	0.27668 (9)	0.96448 (17)	0.0229 (3)	
H10A	0.5467	0.3127	0.8918	0.034*	
H10B	0.7613	0.2534	0.9550	0.034*	
H10C	0.5946	0.3067	1.0456	0.034*	
H1N1	−0.233 (5)	−0.0121 (14)	0.781 (3)	0.031 (6)*	

### Atomic displacement parameters (Å^2^)

**Table:**

	*U*^11^	*U*^22^	*U*^33^	*U*^12^	*U*^13^	*U*^23^
N2	0.0216 (6)	0.0188 (5)	0.0123 (5)	−0.0046 (5)	0.0029 (5)	0.0002 (4)
N1	0.0257 (7)	0.0208 (6)	0.0103 (5)	−0.0085 (5)	0.0011 (5)	−0.0010 (4)
C1	0.0227 (7)	0.0211 (6)	0.0134 (6)	−0.0059 (5)	0.0035 (5)	0.0005 (5)
C2	0.0201 (7)	0.0197 (6)	0.0145 (6)	−0.0052 (5)	0.0007 (5)	−0.0002 (5)
C3	0.0157 (6)	0.0148 (6)	0.0123 (6)	0.0002 (5)	0.0014 (5)	−0.0002 (4)
C4	0.0156 (6)	0.0147 (5)	0.0118 (5)	−0.0008 (4)	0.0004 (4)	0.0003 (4)
C5	0.0251 (8)	0.0204 (6)	0.0111 (6)	−0.0053 (6)	0.0009 (5)	0.0015 (5)
C6	0.0252 (8)	0.0224 (6)	0.0122 (6)	−0.0067 (6)	−0.0025 (6)	−0.0001 (5)
C7	0.0169 (7)	0.0173 (6)	0.0171 (6)	−0.0031 (5)	0.0011 (5)	0.0012 (5)
C8	0.0260 (8)	0.0233 (7)	0.0164 (6)	−0.0081 (6)	0.0076 (6)	−0.0009 (5)
C9	0.0257 (8)	0.0219 (6)	0.0134 (6)	−0.0074 (6)	0.0048 (5)	−0.0024 (5)
C10	0.0211 (8)	0.0226 (6)	0.0249 (7)	−0.0078 (6)	0.0008 (6)	0.0013 (6)

### Geometric parameters (Å, °)

**Table:**

N2—C3	1.2976 (17)	C5—C6	1.391 (2)
N2—C1	1.4763 (19)	C5—H5A	0.9300
N1—C3	1.3627 (17)	C6—C7	1.3975 (19)
N1—C2	1.4641 (19)	C6—H6A	0.9300
N1—H1N1	0.87 (3)	C7—C8	1.389 (2)
C1—C2	1.5447 (19)	C7—C10	1.5092 (19)
C1—H1A	0.9700	C8—C9	1.394 (2)
C1—H1B	0.9700	C8—H8A	0.9300
C2—H2A	0.9700	C9—H9A	0.9300
C2—H2B	0.9700	C10—H10A	0.9600
C3—C4	1.4779 (18)	C10—H10B	0.9600
C4—C5	1.394 (2)	C10—H10C	0.9600
C4—C9	1.397 (2)		
			
C3—N2—C1	106.60 (12)	C6—C5—C4	120.66 (13)
C3—N1—C2	108.04 (12)	C6—C5—H5A	119.7
C3—N1—H1N1	125.8 (17)	C4—C5—H5A	119.7
C2—N1—H1N1	120.4 (18)	C5—C6—C7	120.80 (13)
N2—C1—C2	105.98 (12)	C5—C6—H6A	119.6
N2—C1—H1A	110.5	C7—C6—H6A	119.6
C2—C1—H1A	110.5	C8—C7—C6	118.33 (13)
N2—C1—H1B	110.5	C8—C7—C10	120.66 (13)
C2—C1—H1B	110.5	C6—C7—C10	121.01 (13)
H1A—C1—H1B	108.7	C7—C8—C9	121.21 (13)
N1—C2—C1	101.59 (11)	C7—C8—H8A	119.4
N1—C2—H2A	111.5	C9—C8—H8A	119.4
C1—C2—H2A	111.5	C8—C9—C4	120.25 (14)
N1—C2—H2B	111.5	C8—C9—H9A	119.9
C1—C2—H2B	111.5	C4—C9—H9A	119.9
H2A—C2—H2B	109.3	C7—C10—H10A	109.5
N2—C3—N1	116.31 (12)	C7—C10—H10B	109.5
N2—C3—C4	122.68 (12)	H10A—C10—H10B	109.5
N1—C3—C4	120.98 (12)	C7—C10—H10C	109.5
C5—C4—C9	118.72 (12)	H10A—C10—H10C	109.5
C5—C4—C3	119.75 (13)	H10B—C10—H10C	109.5
C9—C4—C3	121.53 (13)		
			
C3—N2—C1—C2	−5.19 (16)	C9—C4—C5—C6	−1.2 (2)
C3—N1—C2—C1	−11.95 (15)	C3—C4—C5—C6	179.46 (14)
N2—C1—C2—N1	10.31 (15)	C4—C5—C6—C7	0.1 (2)
C1—N2—C3—N1	−2.84 (18)	C5—C6—C7—C8	1.1 (2)
C1—N2—C3—C4	179.35 (13)	C5—C6—C7—C10	−178.05 (14)
C2—N1—C3—N2	10.16 (18)	C6—C7—C8—C9	−1.2 (2)
C2—N1—C3—C4	−171.99 (13)	C10—C7—C8—C9	177.91 (14)
N2—C3—C4—C5	−1.8 (2)	C7—C8—C9—C4	0.2 (2)
N1—C3—C4—C5	−179.56 (14)	C5—C4—C9—C8	1.0 (2)
N2—C3—C4—C9	178.80 (15)	C3—C4—C9—C8	−179.62 (14)
N1—C3—C4—C9	1.1 (2)		

### Hydrogen-bond geometry (Å, °)

**Table:**

*D*—H···*A*	*D*—H	H···*A*	*D*···*A*	*D*—H···*A*
N1—H1N1···N2^i^	0.87 (3)	2.06 (3)	2.9224 (18)	170 (2)
C10—H10B···Cg1^ii^	0.96	2.88	3.8110 (16)	163

Symmetry codes: (i) *x*, −*y*, *z*−1/2; (ii) *x*+1, *y*, *z*.
